# Gastroprotective and Ulcer Healing Effects of Essential Oil of *Hyptis martiusii* Benth. (Lamiaceae)

**DOI:** 10.1371/journal.pone.0084400

**Published:** 2014-01-15

**Authors:** Germana Freire Rocha Caldas, Alisson Rodrigo da Silva Oliveira, Alice Valença Araújo, Dafne Carolina Alves Quixabeira, Jacinto da Costa Silva-Neto, João Henrique Costa-Silva, Irwin Rose Alencar de Menezes, Fabiano Ferreira, Ana Cristina Lima Leite, José Galberto Martins da Costa, Almir Gonçalves Wanderley

**Affiliations:** 1 Department of Pharmaceutical Sciences, Federal University of Pernambuco, Recife, Pernambuco, Brazil; 2 Department of Physiology and Pharmacology, Federal University of Pernambuco, Recife, Pernambuco, Brazil; 3 Department of Histology and Embryology, Federal University of Pernambuco, Recife, Pernambuco, Brazil; 4 Department of Physical Education and Sport Sciences, Federal University of Pernambuco, Vitória de Santo Antão, Pernambuco, Brazil; 5 Department of Biological Chemistry, Regional University of Cariri, Crato, Ceará, Brazil; National Cancer Center, Japan

## Abstract

*Hyptis martiusii* Benth. is an aromatic plant found in abundance in northeastern Brazil that is used in ethnomedicine to treat gastric disorders. The aim of this study was to elucidate the mechanisms of action involved in the gastroprotection of the essential oil of *Hyptis martiusii* (EOHM) and to evaluate its healing capacity. Wistar rats were exposed to different protocols and subsequently were treated with 1% Tween-80 aqueous solution (negative control), pantoprazole, carbenoxolone, N-acetylcysteine (depending on the specificity of each model) or EOHM. The antisecretory activity (basal or stimulated) was determined using the pyloric ligature method. The gastroprotective action of nitric oxide and sulphydryl groups (–SH groups), as well as the quantification of adherent mucus and the levels of malondialdehyde and –SH groups in gastric mucosa, were evaluated using ethanol-induced gastric ulcer model. The healing ability was evaluated using the acetic acid-induced gastric ulcer model and histological and immunohistochemical analysis (HE, PAS and PCNA). EOHM (400 mg/kg) reduced the volume and acidity of gastric secretion stimulated by histamine and pentagastrin. The gastroprotective effect of EOHM involves the participation of endogenous sulfhydryl groups. EOHM increased mucus production (54.8%), reduced levels of MDA (72.5%) and prevented the depletion of –SH groups (73.8%) in the gastric mucosa. The treatment with EOHM reduced in 70.3% the gastric lesion area, promoting significant regeneration of the gastric mucosa, as confirmed by histological analysis and analysis of proliferating cell nuclear antigen. The results show that gastroprotective effect of EOHM is mediated by cytoprotective and antioxidant mechanisms and by their antisecretory activity, and suggest that the essential oil of *Hyptis martiusii* is a promising candidate for the treatment of gastric ulcers.

## Introduction

Despite advances in understanding the etiology, diagnostic modalities and availability of modern treatments for dyspeptic symptoms, peptic ulcer disease and its complications remain a major cause of morbidity and mortality worldwide [1]. A variety of pathogenic mechanisms may contribute to the formation of a peptic ulcer, but, regardless of the etiology, the onset of an ulcer occurs when there is an imbalance in the environment caused by increased aggressive factors of exogenous or endogenous origin, or by decreased gastric resistance, leading to irritation, ulceration and mucosal bleeding [2].

The gastric mucosa uses various defense mechanisms to maintain its integrity against aggression caused by hydrochloric acid, pepsin, bile, NSAID, and other substances. The main mucosal protective factors are mucus, bicarbonate, the prostaglandins, adequate blood flow, somatostatin, nitric oxide, sulfhydryl compounds, motility and cell regeneration [3].

*Hyptis martiusii* Benth. (Lamiaceae) is an aromatic plant found in abundance in northeastern Brazil, where it is popularly known as *cidreira-do-mato* or *cidreira-brava* and is known as a potential source of essential oils, like other species of the genus Hyptis. The medical uses of some species, such as *Hyptis suaveolens, Hyptis pectinata*, *Hyptis crenata* and *Hyptis fruticosa* are well-known among the population. According to ethnopharmacological studies, an infusion or decoction of leaves of *Hytpis martiusii* are used against diseases of the stomach and intestine [4].

Few investigations of the pharmacological properties of *Hyptis martiusii* are described in the literature, including cytotoxic and antiproliferative effects on certain tumor cell lines [5], [6], insecticidal activity against larvae of *Aedes aegypti* and *Culex quinquefasciantus* [7], antimicrobial activity against resistant strains of *Staphylococcus aureus* and *Escherichia coli* [8] and antioxidant activity [9]. In addition, our laboratory previously reported the antiulcerogenic activity in models of acute gastric ulcers and potent antisecretory activity in the essential oil of *Hyptis martiusii* leaves [10]. We thus investigated the action mechanisms involved in the gastroprotective effects of the essential oil of *Hyptis martiusii* (EOHM) and its ulcer healing properties.

## Materials and Methods

### Plant material

*Hyptis martiusii* (Lamiaceae) leaves were collected on the Araripe Plateau, in Crato, in the Brazilian State of Ceará (S 7°21.744′–W 39°28.691′). Entire plants were collected during the flowering stage, in June 2012. The access of the material was authorized by the Biodiversity Authorization and Information System from the Ministry of Environment of Brazil (document number 33635-1). A representative sample of this species is deposited in the Prisco Bezerra Herbarium of the Department of Biology at the Federal University of Ceará (UFC) (registration number 43038).

### Extraction of essential oil

The leaves were dried at room temperature for 72 h prior to hydrodistillation and the essential oil was extracted immediately thereafter. Three portions (780.70±12.13 g) of the dried leaves were individually subjected to hydrodistillation using a Clevenger-type apparatus for 3 h. The yield of the essential oil from dried leaves of *Hyptis martiusii* was 0.97±0.01% (w/w), corresponding to 7.59±0.05 g of oil, calculated according to the mean dry weight of the leaves used in each extraction. The water/oil mixture was collected, the aqueous solution was discarded, the oil was dried over anhydrous sodium sulfate and then filtered. Essential oil was stored in an amber bottle at −20°C ready for pharmacological experiments and phytochemical analysis.

### Identification of the constituents of essential oil

The EOHM analysis was performed in a gas chromatographer attached to a mass spectrometer (GC-MS, SHIMADZU QP5050A) equipped with a capillary column (DB–5HT, 30 m×0.25 mm, 0.1 µm film thickness) with the following specifications: helium as carrier gas (1.0 mL/min flow rate); injector temperature 270°C and detector temperature 290°C; linear velocity of 47.3 cm/sec; pressure of 107.8 kPa; column temperature programmed from 60°C (2 min) to 180°C (1 min) at 4°C/min, then from 180 to 260°C at 10°C/min (10 min). The mass spectrometer was operated using 70 eV of ionization energy. Identification of individual constituents was based on the interpretation of their mass spectral fragmentation using computer-based library MS standard searches (Wiley 229), retention indices and comparison with the mass spectral database and data from the literature [11].

### Animals

Male and female Wistar rats (200–300 g) were obtained from the Department of Physiology and Pharmacology of Federal University of Pernambuco (UFPE), Pernambuco, Brazil. These were kept under standard environmental conditions (12 h dark/light cycle) and temperature (22±2°C). Water and industrialized dry food (Labina®, Purina, Brazil) were made available *ad libitum*. All the experimental protocols were submitted to and approved by the Animal Experimentation Ethics Committee of the Federal University of Pernambuco (UFPE), license n°. 012490, in accordance with the National Institute of Health's Guide to the Care and Use of Laboratory Animals.

### Reagents and chemicals

The following substances were used: sodium acetate, Alcian Blue, atropine, thiobarbituric acid, trichloroacetic acid, 5,5′-dithiobis (2-nitrobenzoic acid), bethanechol, carbenoxolone, 2,2-diphenyl-1-picrylhydrazyl, EDTA, glutathione, histamine, N-acetylcysteine, N-ethylmaleimide, nitro_ω_-L-arginine methyl ester, pantoprazole, pentagastrin, ranitidine, sodium lauryl sulfate, thymol, 1,1,3,3-tetramethoxypropane (Sigma, St. Louis, USA), tris (hydroxymethyl) aminomethane, acetic acid, hydrochloric acid, ethanol, n-butanol, magnesium chloride, sodium chloride, potassium chloride, glucose, sodium hydroxide, anhydrous sodium sulfate, polysorbate 80 - Tween 80 (Vetec, Duque de Caxias, Brazil), ethyl ether, formaldehyde, phenolphthalein (FMaia, Cotia, Brazil), xylazine, ketamine (Vetbrands, Paulinia, Brazil). For the purposes of the experiment, the essential oil of *Hyptis martiusii* was emulsified in a Tween 80 at 1% before administration to the animals.

### Evaluation of mucosal protective factors

Each experimental model comprised the following groups: positive control (pantoprazole – a proton pump inhibitor, carbenoxolone - a cytoprotective agent or N-acetylcysteine – the standard antioxidant drug) depending on the specificity of each model; a negative control (1% Tween-80 aqueous solution) or EOHM. Previous pharmacological studies performed by our group on the essential oil of dried leaves of *Hyptis martiusii* showed antiulcerogenic activity at doses of 100, 200 and 400 mg/kg in acute gastric ulcer models. The dose of 400 mg/kg was chosen for additional studies in order to shed light on the mechanisms underlying its gastroprotective effect, as this had been shown to be the most effective dose in previously assessed protocols.

### Determination of gastric acid secretion stimulated with histamine, bethanechol and pentagastrin

The experiment was carried out using the pyloric ligature method described by Shay et al. [12], with slight modifications. The animals were divided into 10 groups (n = 6): (1) control, (2) EOHM, (3) histamine, (4) histamine plus ranitidine, (5) histamine plus EOHM (6) bethanechol, (7) bethanechol plus atropine, (8) bethanechol plus EOHM, (9) pentagastrin or (10) pentagastrin plus EOHM. They were fasted for 16 h with free access to 5% glucose solution. For pyloric ligature method, the animals were anaesthetized (xylazine, 6 mg/kg and ketamine, 60 mg/kg, intraperitoneally) and immediately after ligature they received an intraduodenal dose of EOHM (400 mg/kg), a control (1% Tween-80 aqueous solution, 0.1 mL/100 g body weight), ranitidine (60 mg/kg) or subcutaneous atropine (1 mg/kg). The abdominal wall was sutured and, 1 h after pylorus ligation, the animals received subcutaneously histamine (20 mg/kg), bethanechol (2.5 mg/kg) or pentagastrin (400 µg/kg) stimulus. Four hours after pylorus ligation, the animals were sacrificed, the gastric secretion collected and centrifuged at 176× g for 30 min. The volume (mL), pH values and the total acidity (mEquiv.[H^+^]/mL/4 h) were determined.

### Determination of gastric mucus

Adherence to the gastric wall mucus was quantified using the method described by Corne et al. [13] using the ethanol-induced ulcer model [14]. After fasting for 16 h, the animals (n = 6) were treated with 1% Tween-80 aqueous solution (CL), pantoprazole (40 mg/kg) or EOHM (400 mg/kg) 1 h before ethanol (70%, 0.5 mL/100 g, p.o.) was used to induce a gastric lesion. The non-injured control group (CN) received no treatment. The animals were sacrificed 1 h after the administration of ethanol and their stomachs were removed. Each glandular segment was weighed and immediately transferred to a tube containing 10 mL of 0.1% Alcian Blue and stained for 2 h. The dye complexed to the mucus gland wall was extracted with 10 mL of magnesium chloride (0.5 mol/L) and agitated for 2 h. At 4 mL of the mixture, 4 mL of diethyl ether were added and the solution was shaken. The emulsion obtained was centrifuged at 1480× g for 10 min. The absorbance of samples was read at 598 nm and results were expressed as µg of Alcian Blue/g of tissue.

### Determination of the role of nitric oxide (NO) and sulfhydryl groups (–SH) in gastroprotection

To investigate the influence of endogenous NO and –SH groups on the gastroprotective effect, the animals fasted for 24 h and were divided into nine groups (n = 6) of which three were intraperitoneally pretreated with saline (solution of 0.9% w/v of sodium chloride), three with L-NAME (N_ω_-nitro-L-arginine methyl ester, 70 mg/kg, intraperitoneally), an inhibitor of the NO-synthase enzyme and three with NEM (N-ethylmaleimide, 10 mg/kg, intraperitoneally), a blocker of sulfhydryl compounds [15], [16]. 30 min after pretreatment, each group received an oral administration of 1% Tween-80 aqueous solution (control), carbenoxolone (100 mg/kg) or EOHM (400 mg/kg). After 1 h, all the animals received 1 mL of absolute ethanol (oral via) to induce gastric lesions. The animals were sacrificed after 1 h of ethanol administration, the stomachs were removed for determination of gastric lesions as previously described.

### In vitro study of radical scavenging activity – DPPH assay

The free radical scavenging ability of the EOHM and 1,8-cineole was evaluated using a modified version of the DPPH method (2,2-diphenyl-1-picryl-hydrazyl) [17]. Samples were prepared in triplicate using aliquots of 3 mL of ethanolic solution of DPPH (40 µg/mL) and 1 mL of ethanol solution containing different concentrations (0.3–3.0 mg/mL) of EOHM, 1,8-cineole or positive control (thymol). The solutions were mixed and incubated for 30 min at room temperature and the absorbance was read at 517 nm. The IC_50_ (inhibitory concentration of sample required to reduce the absorbance of the negative control by 50%) was calculated from a calibration curve obtained from the % of antioxidant activity versus concentration of EOHM, 1,8-cineole or thymol. Equation for antioxidant activity: % antioxidant activity = Abs negative control - (Abs sample - Abs blank)×100/Abs negative control.

### In vivo antioxidant activity

The antioxidant tests were performed with the homogenate of the gastric mucosa of animals with ethanol-induced ulcers [14]. The animals were divided into four groups (n = 6). After fasting for 16 h, the animals were treated orally with 1% Tween-80 aqueous solution (CL), N-acetylcysteine (NAC, 750 mg/kg) or EOHM (400 mg/kg) 1 h before administration of the ulcerogenic agent. Gastric lesions were induced using ethanol (70%, 0.5 mL/100 g, oral via). The animals were sacrificed 1 h after the administration of ethanol, their stomachs were removed and opened along the great curvature. The uninjured control group consisted of untreated animals, exposed to experimental procedures, but without effective ulcer induction.

### Determination of lipid peroxidation (LPO)

The lipid peroxidation index was determined using the method described by Ohkawa et al. [18] involving measuring malondialdehyde (MDA). The stomach tissue excised was homogenized in a cold KCl (0.15 mol/L) solution and centrifuged at 11,000× g for 20 min at 4°C. Aliquots of 0.2 mL of sodium lauryl sulfate (8.1%), 1.5 mL of acetic acid (20%, pH 3.5), 1.5 mL of thiobarbituric acid (0.8%, w/v) and 0.3 mL of distilled water were added to 0.5 mL of the homogenate. The samples were incubated in a water bath at a temperature of 95°C for 1 h. After cooling, 6 mL of an n-butanol+distilled water mixture (5∶1, v/v) was added, the tubes were vortexed for 1 min, and finally centrifuged at 1073× g for 10 min. The absorbance was measured at 532 nm and the results were expressed as µmol of MDA/g tissue.

### Quantification of non-protein sulfhydryl groups (–SH groups)

The levels of non-protein sulfhydryl groups (–SH groups) in the gastric mucosa were determined using the method developed by Sedlak and Lindsay [19]. The excised stomach tissue was weighed and homogenized in a cold EDTA (0.02 mol/L) solution. Aliquots of 320 µL of distilled water and 80 µL 50% aqueous solution of trichloroacetic acid were added to 400 µL of the homogenate for protein precipitation and the samples were then centrifuged at 604× g for 15 min at 4°C. To a total of 400 µL of supernatant was added 800 µL of 0.4 M Tris (pH 8.9) and 20 µL of 5,5-dithiobis(2-nitrobenzoic acid) to 0.01 M. The mixture was then stirred for 3 min and the absorbance was measured at 412 nm. The concentrations of non-protein sulfhydryl groups were expressed in µg of GSH/g tissue.

### Evaluation of healing properties

#### Acetic acid-induced gastric ulcer

Chronic ulcer induction was based on described by Takagi et al. [20] with some modifications. The animals were divided into 3 groups (n = 6), given a restricted solid food diet for 24 h and, after this, anesthetized in order to perform surgery to expose the stomach. 0.05 mL of 30% acetic acid was injected into the subserosal layer of the external wall of the stomach. One day after administration of acid, daily treatment began and the animals were treated orally once daily for 14 days with 1% Tween-80 aqueous solution (control), pantoprazole (40 mg/kg) or EOHM (400 mg/kg). During this period, the possible toxic effects of EOHM were evaluated using such parameters as mortality, changes in body mass and macroscopic analysis of vital organs. On day 15, all groups were sacrificed, the stomachs removed, photographed and the surface area of gastric lesion determined by computerized planimetry (Software ImageJ®) and the data expressed in mm^2^.

### Histological analysis

The stomach lesions induced by acetic acid in rats undergoing different treatments were located, sectioned, and set in 10% buffered formalin. After setting, the samples was washed with water, immersed in 70% ethyl alcohol for 3–4 days and embedded in paraffin. Five-µm thick paraffin sections were taken and stained with hematoxylin/eosin (HE) and Periodic Acid–Schiff (PAS). Histological analysis of the gastric sections was carried out using an automatic microscopy system MICRO DIP® (Kacil Inc.).

### Immunohistochemical analysis

The immunohistochemical for proliferating cell nuclear antigen (PCNA) was performed in samples of rats' stomachs embedded in paraffin. Sections of 4 µm were obtained and incubated for 30 min with monoclonal antibody against the anti-PCNA protein. Initially, the samples were deparaffinized in xylene and hydrated. Then antigenic retrieval was performed in microwave oven at 100°C, the slides were cooled to room temperature and endogenous peroxidase was blocked by the incubation in peroxidase blocking solution for 7.5 min. After cooling, the slides were incubated separately with primary antibodies for PCNA (anti-PCNA antibody [PC10] - Proliferation Marker (ab29) - Mouse monoclonal antibody, Abcan Inc), 1∶100, 30 min, and with secondary antibody (Nichirei Biosciences Inc.), 1∶200 for 30 min and then washed with phosphate buffered saline (PBS). After washing, slides were incubated with diaminobenzidine chromogen solution (DAB), washed in water, counter-stained with hematoxylin, dehydrated and mounted. Cells reactive for anti-PCNA were identified by the presence of a dark reddish-brown chromogen in the nucleus of epithelial cells. The reactivity was indicated using the following scores: mild, moderate and strong reactivity.

### Statistical analysis

Values were expressed as mean ± standard error of mean (S.E.M.). The differences between groups were determined by analysis of variance (ANOVA) followed by Tukey's test. Statistical analysis was performed using GraphPad Prism 5.0®. The level of significance for rejection of the null hypothesis was set at 5% (*p*<0.05).

## Results

### Chemical analysis of essential oil

Chemical characterization of the EOHM using GC-MS identified 26 components, accounting for 95.52% of the total oil. The main components identified in the essential oils of *Hytpis martiusii* were 1,8-cineole (32.80%), δ-3-carene (17.43%), camphor (6.70%), α-pinene (3.52%) and caryophyllene oxide (3.50%). Table 1 shows the constituents identified and the percentage composition according to their retention times.

**Table 1:** Chemical constituents of essential oil of leaves of *Hyptis martiusii* Benth.

Components	Retention Time (min)	(%)
Hexen-1-ol	4.39	1.81
*α*-Pinene	6.59	3.52
*β*-Pinene	8.18	2.28
*β*-Myrcene	8.69	1.81
δ-3-Carene	9.60	17.43
*p*-Cymene	10.01	0.87
*o*-Cymene	10.35	3.36
1,8-Cineole	10.76	32.80
Linalool	13.89	1.21
Camphor	16.31	6.70
Isoborneol	17.54	0.99
*trans*-Caryophyllene	30.69	3.37
Aromadendrene	31.63	1.96
α-Humulene	32.51	1.69
Ledene	34.32	0.99
Germacrene B	34.59	2.21
γ-Selinen	36.58	0.77
β-Panasinsene	36.81	0.97
Isolongifol	37.74	0.97
Palustrol	38.71	0.83
Spathulenol	38.62	1.85
Caryophyllene oxide	38.82	3.50
Globulol	38.98	0.91
Ledol	39.85	1.04
Rosifoliol	40.04	0.86
(*Z*)-Valerenyl acetate	42.43	0.82
**Total**		**95.52**

### Effect of the EOHM on stimulated gastric acid secretion

It was found that, after 4 h of ligation of the pylorus, intraduodenal administration of EOHM reduced the volume of gastric secretion, increased the pH of gastric juices and reduced the total acidity of the acid secretion compared to controls not stimulated by secretagogues. Histamine, pentagastrin and bethanechol, when administered subcutaneously, stimulated base gastric acid secretion, increasing the volume and total acidity and decreasing the pH of gastric acid. Ranitidine (60 mg/kg) and atropine (1 mg/kg) prevented the increase of the volume and acidity, as well as the decrease of the pH of gastric acid secretion stimulated by histamine and bethanechol, respectively. The EOHM was able to prevent the increase in volume and acidity of gastric acid secretion stimulated by histamine and pentagastrin, but was not able to change the gastric acid secretion parameters affected by bethanechol (Table 2).

**Table 2:** Effect of the essential oil of *Hyptis martiusii* (EOHM) on gastric secretion parameters basal or stimulated by histamine (20 mg/kg), bethanechol (2.5 mg/kg) or pentagastrin (400 µg/kg) in Wistar rats subjected to pylorus ligature.

Stimulus+treatment	Gastric volume (mL)	pH	Total acidity (mEquiv. [H^+^]/mL/4 h)
control (not estimulated)	4.4±0.7	1.6±0.2	42.5±11.6
EOHM (not estimulted)	1.8±0.2*	2.4±0.2*	9.2±1.7*
histamine	6.3±0.7	1.5±0.0	52.8±9.9
histamine+ranitidine	2.3±0.3#	2.6±0.1#	2.9±0.6#
histamine+EOHM	4.2±0.2#	1.8±0.1	24.8±2.9#
bethanechol	7.8±0.5	1.5±0.0	52.2±5.4
bethanechol+atropine	2.8±0.2##	2.5±0.2##	6.6±1.5##
bethanechol+EOHM	6.3±0.2	1.6±0.0	38.8±5.3
pentagastrin	7.1±1.4	1.4±0.0	45.0±7.8
pentagastrin+EOHM	3.4±0.2###	1.91±0.1	14.6±3.4###

Values are expressed as mean ± S.E.M. Treatment: control (C, 1% Tween-80 aqueous solution, 0.1 mL/100 g, i.d), EOHM (400 mg/kg, i.d), ranitidine (60 mg/kg, i.d.) and atropine (1 mg/kg, s.c).

*p*<0.05 vs. control group,

^#^ *p*<0.05 vs. histamine group,

## *p*<0.05 vs. bethanechol group and

### *p*<0.05 vs. pentagastrin group (ANOVA followed by Tukey's test).

### Evaluation of mucosal protective factors

#### Effect of the EOHM on the production of gastric mucus

**Figure 1:**
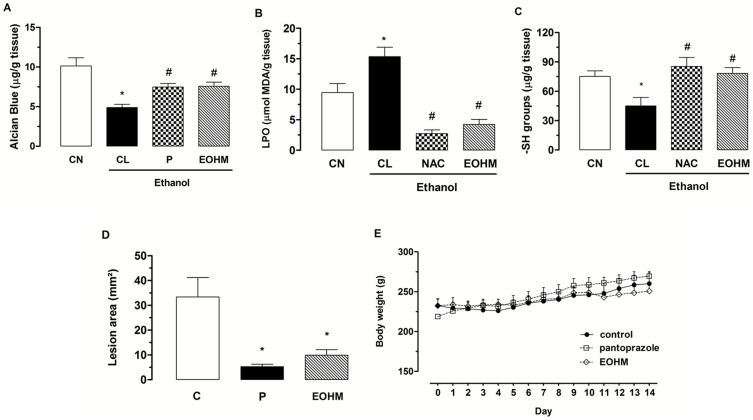
Effect of the essential oil of *Hyptis martiusii* (EOHM) on quantification of mucus (A), levels of malondialdehyde (B) and sulfhydryl groups (C) in the gastric ulcers model induced by ethanol (70%, 0.5 mL/100 g), in the healing ulcer (D) and body weight of rats (E) after formation of chronic ulcer induced by 30% acetic acid. The non-injured control group (CN) received no treatment. The experimental groups received 1% Tween-80 aqueous solution (CL or C, injured control), pantoprazole (P, 40 mg/kg), N-acetylcysteine (NAC, 750 mg/kg) or EOHM (400 mg/kg). Results are expressed as mean ± S. E. M. ANOVA followed by Tukey's test (*p<0.05 vs. non-injured control group-CN and ^#^ *p*<0.05 vs. injured control group-CL).

Figure 1A shows that the animals injured with ethanol (CL) showed a significant decrease in the levels of gastric mucus (4.9±0.4 µg of Alcian Blue/g of tissue) compared to the non-injured control group (CN, 10.1±1.0 µg of Alcian Blue/g of tissue). The treatment with EOHM at a dose of 400 mg/kg was capable of increasing mucus production to a significant degree (7.6±0.5 µg of Alcian Blue/g of tissue) compared to the injured group that received 1% Tween-80 aqueous solution. Pantoprazole, used as positive control, also caused a significant increase in the levels of gastric mucus (7.5±0.4 µg of Alcian Blue/g of tissue).

#### Role of nitric oxide (NO) and sulfhydryl groups (–SH) in EOHM gastroprotection

Both the NO-synthase inhibitor, L-NAME (N_ω_-nitro-L-arginine methyl ester) and the blocker of sulfhydryl compounds, NEM (N-ethylmaleimide), caused an increase in gastric lesions in all groups compared to the groups pretreated with saline. In animals pretreated with saline, treatment with EOHM (400 mg/kg) had a gastroprotective effect as expected, as the oil inhibits the formation of gastric lesions induced by ethanol. In rats pretreated with L-NAME, EOHM also had a gastroprotective effect. However, the depletion of sulfhydryl groups by pretreatment with NEM was able to significantly reduce the gastroprotective effect of EOHM (Table 3).

**Table 3:** Effect of oral administration of essential oil of *Hyptis martiusii* (EOHM) on gastric lesions induced by ethanol in Wistar rats pretreated with L-NAME (N_ω_-nitro-L-arginine methyl ester, 70 mg/kg) or NEM (N-ethylmaleimide, 10 mg/kg).

Pretreatment	Treatment (p.o.)	Dose (mg/kg)	Lesion area (mm^2^)	Inhibition (%)
Saline (i.p.)	control	-	316.9±49.9	-
	carbenoxolone	100	6.6±2.9*	97.9
	EOHM	400	5.8±5.4*	98.1
L-NAME (i.p.)	control	-	655.3±51.2*	-
	carbenoxolone	100	228.5±76.3#	65.1
	EOHM	400	6.5±4.0#	99.0
NEM (i.p.)	control	-	563.0±1.3*	-
	carbenoxolone	100	380.0±42.4	32.5
	EOHM	400	427.6±59.3	24.0

Results are expressed as mean ± S.E.M.

* *p*<0.05 compared to saline+control,

# *p*<0.05 compared to L-NAME+control (ANOVA followed by Tukey's test).

### DPPH free-radical scavenging assay

The results show that the essential oil of *Hyptis martiusii* (EOHM) exhibited no significant relative ability to promote the capture of the DPPH radical at any of the concentrations tested (0.3–3.0 mg/mL), since the IC_50_ (15.78±0.99 mg/mL) was very high. The 1,8-cineole, a major component of the essential oil, was likewise unable to promote the capture of DPPH. It was not possible to calculate the IC_50_. Thymol, a monoterpene common in essential oils, was used as a reference compound since it has known antioxidant properties and an IC_50_ of 0.67±0.02 mg/mL [21].

### Effects of the EOHM on the antioxidant activity system

The rate of lipid peroxidation (LPO) in gastric mucosa of rats subjected to ethanol-induced gastric ulcer was determined by quantifying malondialdehyde, which reacts with thiobarbituric acid. Animals in the control group showed an increase in gastric levels of malondialdehyde in injured rats (15.3±1.5 µmol MDA/g of tissue) compared to the uninjured control group that received only 1% Tween-80 aqueous solution (9.4±1.5 µmol of MDA/g of tissue). Treatment with EOHM (400 mg/kg) decreased the rate of lipid peroxidation by significantly diminishing the production of malondialdehyde produced by ethanol (4.2±0.8 µmol MDA/g of tissue). Oral treatment with N-acetylcysteine (NAC, 750 mg/kg) also inhibited the increase in the levels of malondialdehyde (2.7±0.6 µmol of MDA/g of tissue) (Figure 1B).

**Figure 2:**
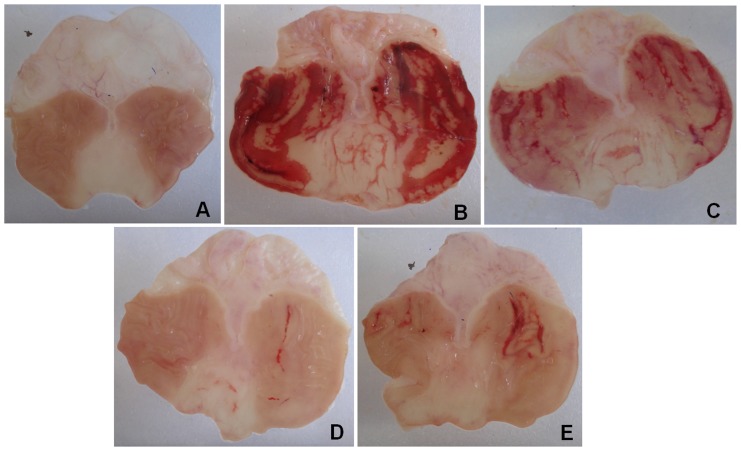
Effect of the essential oil of *Hyptis martiusii* (EOHM) on the macroscopical appearance of the gastric mucosa in rats after the induction of gastric lesions by ethanol (70%, 0.5 mL/100 g, p.o). The non-injured control group (A) received no treatment. (B) injured control (1% Tween-80 aqueous solution), (C) pantoprazole (40 mg/kg), (D) N-acetylcysteine (750 mg/kg) or (E) EOHM (400 mg/kg).

The levels of sulfhydryl groups (–SH groups) in the gastric mucosa of the uninjured animals of the control group were 75.1±5.7 µg/g of tissue, but in animals with an ethanol-induced gastric lesion (injured control) a reduction in the levels of GSH in the glandular region of the gastric mucosa (45.0±8.7 µg/g of tissue) was observed, compared to baseline levels in the uninjured control group. Treatment with N-acetylcysteine (NAC, 750 mg/kg) and EOHM (400 mg/kg) showed that both were capable of reversing the reduction in levels of sulfhydryl groups in the mucosa, returning the antioxidant system to base levels (85.3±9.2 and 78.2±6.0 µg/g of tissue), respectively (Figure 1C). Figure 2 shows the effect of the essential oil of *Hyptis martiusii* on the macroscopic appearance of the gastric mucosa in ethanol-induced gastric mucosal lesions.

### Evaluation of the healing properties of EOHM

#### Effect of EOHM on acetic acid-induced gastric ulcer

Oral administration of EOHM (400 mg/kg) for 14 consecutive days of treatment, significantly decreased (70.3%) the area of chronic ulcer to 9.9±2.2 mm^2^ compared to the control group treated with 1% Tween-80 aqueous solution (control), in which the injured area corresponded to 33.3±7.8 mm^2^ (Figure 1D). Pantoprazole (40 mg/kg) speeded up the healing of gastric ulcer, reducing the area of the lesion to a statistically significant extent by 5.2±1.0 mm^2^ (82.7%) compared to the control group. There were no visible signs of toxicity (diarrhea or changes in behavior or locomotor activity) in animals treated with EOHM and pantoprazole for 14 days, the animals treated with the essential oil showed body weight gain (Figure 1E) and organ weight (data not shown) similar to those of animals in the control group.

### Histological analysis

**Figure 3:**
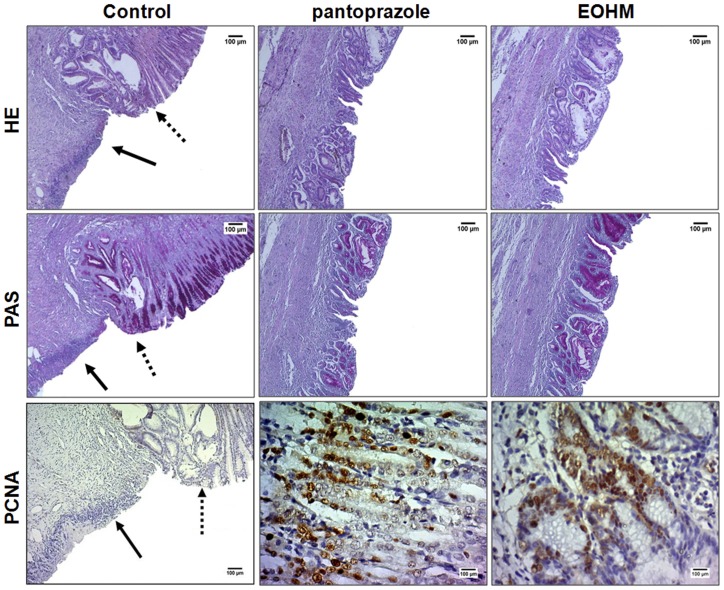
Histological study and immunohistochemical analysis for PCNA (proliferating cell nuclear antigen) of gastric mucosa of rats treated with 1% Tween-80 aqueous solution (control), pantoprazole (40 mg/kg) and essential oil of *Hyptis martiusii* (EOHM, 400 mg/kg) for 14 days after injury induced by 30% acetic acid. The filled arrow indicates the absence of the epithelial layer (ulcer area internal) and the dashed arrow indicates epithelial layer remaining (ulcer edge). Haematoxylin/eosin (HE) and Periodic Acid–Schiff staining (PAS), magnification, 40×. Microphotographs depict PCNA immunoreactivity in the groups, magnification, 200× (control) and 500× (pantoprazole or EOHM).

The HE staining revealed the presence of an ulcer penetrating the wall caused by gastric mucosa damage induced by 30% acetic acid and confirmed the gastroprotective action of EOHM (400 mg/kg) or pantoprazole (40 mg/kg) after 14 days of treatment, with significant regeneration of the gastric mucosa. PAS staining also revealed the presence of gastric mucus and the arrangement of intact glands in the groups treated with pantoprazole or EOHM. Figure 3 shows the lesion regeneration area (HE) and the great amount of mucus secretion (PAS) evidenced by the intense tone of pink in the pantoprazole and EOHM groups

### Immunohistochemical analysis

The gastric tissues obtained in the acetic acid-induced gastric ulcer model were used for immunohistochemical localization of PCNA antibody. In Figure 3, the PCNA-positive nuclei are marked by reaction with the color brown. In the control group, it was observed the absence of reactivity due to the destruction of the epithelial layer, but it is possible to observe a moderate activity in the layer adjacent to the ulcer. In animals treated with pantoprazole or EOHM, it was observed intense reactivity, with a greater number of labeled cells, with PCNA-positive nuclei marked. The EOHM group showed a higher percentage of labeled nuclei, suggesting that the treatment promotes an expressive increase in cell proliferation in the area of gastric mucosal healing.

## Discussion

In this study we investigated the cytoprotective mechanisms that may be involved in the gastroprotective effect of the essential oil of *Hyptis martiusii*, such as its effect on mucus production, the role of nitric oxide and sulfhydryl groups, the possible antioxidant activity and its healing action regarding acetic acid-induced chronic ulcer. We also tried to clarify the possible mechanism of action responsible for the inhibition of gastric acid effect of EOHM.

Data from the literature have demonstrated experimentally that a large number of essential oils of aromatic and medicinal plants possess gastroprotective and ulcer healing properties [22]. In essential oils in general, the major constituents are monoterpenes and sesquiterpenes, and this applies to species of the genus *Hyptis* where these constituents are identified as the main components [23]. Confirming these data, the chemical characterization of the essential oil of *Hyptis martiusii* identified 26 components and between monoterpenes and sesquiterpenes the main constituents were 1,8-cineole, δ-3-carene, camphor, α-pinene and caryophyllene oxide.

In a study previously reported by us [10], the phytochemical characterization of the essential oil identified 24 components, presenting as major components bicyclogermacrene, trans-caryophyllene, caryophyllene oxide, 1,8-cineole, δ-3-carene and ledene. In the current analysis, we have observed differences in the quantity, chemical composition and yield of these same constituents. For example, there was no bicyclogermacrene and the percentage of various constituents. Such variations may be due to environmental factors, such as temperature, humidity, exposure to sun, wind regimes, and others factors that may affect chemical composition of the essential oils. Despite the differences between the chemical composition, the essential oil of *Hyptis martiusii* continued exerting its gastroprotective effect. Moreover, since the essential oil is a complex mixture of chemical compounds, we cannot ensure that the gastroprotective activity is associated with a specific chemical compound, but it is possibly a synergic effect of the various components.

Recently, data from our laboratory showed that the essential oil of *Hyptis martiusii* leaves displayed a significant gastroprotective effect on different models of gastric lesions in Wistar rats [10]. In this same study, the intraduodenal administration of the essential oil was able to decrease basal acid secretion in the gastric mucosa in rats with the pyloric ligation model, providing significant antisecretory activity.

In order to identify the effect of the EOHM on the receptors/mediators of the parietal cell in the gastric mucosa, we used the same technique of pyloric ligature, but the gastric acid secretion was stimulated with the agonists (secretagogues) of receptors of histamine (H_2_), acetylcholine (M_3_) and gastrin (CCK_2_). EOHM exhibited inhibitory action regarding gastric acid secretion, interfering with the volume and acidity of secretion induced by stimulation with histamine in the H_2_ receptor and with pentagastrin in the CCK_2_ receptor, but with no effect on gastric acid secretion induced by bethanechol in muscarinic receptors. These results suggest that the decrease in the volume and acidity of secretion in animals treated with EOHM is through interactions of compounds in connection with oil/signaling mediated by the histamine H_2_ and gastrin CCK_2_ receptors.

The first line of defense against acid is the mucus, which together with bicarbonate, covers the entire gastric mucosa and protects against bacterial colonization and mechanical forces of proteolytic digestion. The mucus acts as an antioxidant, reducing the damage caused by free radicals and lubricant gastric surface [24]. The results show a statistically significant increase in the amount of mucus adhering to the gastric mucosa in animals treated with EOHM, thereby explaining the gastroprotective action observed previously.

Nitric oxide (NO) is synthesized by NO-synthase (NOS) from oxygen (O_2_) and L-arginine and, owing to its ability to increase blood flow in gastric mucosa, to regulate mucus production and inhibit the attachment of neutrophils to endothelial cells, has been described as an important modulator of the integrity of the gastric mucosa, along with endogenous PGs [25]. In order to establish the involvement of NO in the gastroprotective effect of EOHM an NO synthase inhibitor (L-NAME) was used and it was found that in animals pretreated with L-NAME, EOHM continued to exert a gastroprotective effect, possibly suggesting that this effect is not dependent on NO release/synthesis.

The endogenous non-protein sulfhydryl groups (–SH groups) present in the mucus and some enzymes of the antioxidant system are directly involved in protection of the gastric mucosa, since they participate in the production of gastric mucus and bind to the free radicals formed during inflammation or produced after exposure of the mucosa to harmful agents, performing a neutralizing function [26]. The participation of sulfhydryl groups in gastric protection provided by EOHM was assessed by pretreatment of animals with an inhibitor of –SH compounds (NEM). The decrease in these groups caused by NEM was able to reduce the gastroprotective effect of EOHM to a statistically significant extent, suggesting the participation of sulfhydryl groups and that the protective effect is dependent on the presence of these groups.

Although some terpene compounds present in essential oils have been described as antioxidants and antioxidant activity (IC_50_ = 0.13 mg/mL) has already been reported for the ethanol extract of this species [9], in the present study, the DPPH method revealed no such activity with the essential oil of *Hyptis martiusii*. A similar response was observed for other species of the same genus, Rebelo et al. [27] reported that the methanol extract of *Hyptis crenata* showed significant antioxidant activity (IC_50_ = 0.01 mg/mL) while the essential oil of leaves did not (IC_50_ = 6.88 mg/mL). It is possible that the absence of antioxidant activity of the oil is related to the fact that its major compound 1,8-cineole, did not show such activity. These data corroborate the results obtained by Amakura et al. [28] and Ojeda-Sana et al. [29] in which it was not possible to infer an IC_50_ value for 1,8-cineole.

The fact that EOHM does not exhibit in vitro antioxidant activity, when using the DPPH method, does not mean that it will not show an antioxidant profile in other types of tests of the same activity. We thus evaluated the action of EOHM on oxidative stress caused by ethanol-induced gastric damage in rats, to show possible antioxidant activity. Ethanol destroys cells by causing mucosal disturbances in the microcirculation of free radicals in the mucosa, increased lipid peroxidation, a decrease of non-protein sulfhydryl groups (GSH) and mucus production and inhibition of gastric prostaglandins [30]. Furthermore, ethanol also destroys epithelial cells in the stomach, causing infiltration of inflammatory cells such as neutrophils and macrophages, which eventually produce vasoconstriction, submucosal edema and hemorrhagic lesions [31].

Confirming data already reported in the literature, we found increased levels of malondialdehyde (MDA) and a decrease in non-protein sulfhydryl groups (–SH groups) in stomachs with ethanol-induced gastric damage, compared to the levels found in non-injured animals. As expected, N-acetylcysteine, an antioxidant, inhibited the increase in MDA levels and abolished the decrease in –SH groups. Lipid peroxidation occurs when reactive oxygen species attack cell membranes, allowing them to enter intracellular structures. Malondialdehyde (MDA) appears as an end product of fatty acid oxidation and the higher the concentration, the higher the level of substances that react with thiobarbituric acid, which is indicative of increased lipid peroxidation [32]. Pretreatment with EOHM (400 mg/kg) significantly reduced lipid peroxidation, as evidenced by reduced levels of malondialdehyde. In view of previous reports regarding sulfhydryl groups, we can say that they are directly associated with maintaining the integrity of the gastric mucosa. Quantification of mucosal –SH groups revealed that EOHM significantly increased basal levels of –SH groups, confirming the involvement of these groups in the gastroprotective effect.

Other essential oils from species of the same genus, such as *Hyptis spicigera* and *Hyptis crenata*, have very similar chemical composition of the essential oil of *Hyptis martiusii*, as well as have demonstrate a gastroprotective activity. According to Takayama et al. [33], the essential oil from *H. spicigera* Lam. provided effective gastroprotection in models of experimental gastric ulcer induced by ethanol or indomethacin, and this effect does not depend on nitric oxide or sulfhydryl groups. They also reported that it does not interfere with H^+^ secretion in gastric mucosa. Interestingly, our results confirm the inhibitory effect of EOHM on the secretion of H^+^ in the gastric mucosa and demonstrate that the gastroprotective effect does not depend on nitric oxide, but it is dependent on the presence of sulfhydryl groups.

In a study recently described by Diniz et al. [34], the essential oil of *H. crenata* Pohl ex Benth. promoted a significant inhibition of gastric lesions induced by ethanol or indomethacin, as well as it has produced a decrease in lipid peroxidation. Furthermore, the oil decreased the gastric emptying, but did not alter the intestinal transit ratio. Similar results have been found in our previous study [10], in which we have demonstrated that EOHM inhibits the formation of ulcers and reduces the gastric emptying, but did not show any effect on intestinal transit. In addition, in this study, we verified that the EOHM have reduced lipid peroxidation.

The administration of acetic acid to the gastric mucosa of rats is capable of producing a well-defined lesion and delaying the healing of wounds, similar to peptic ulcers in humans [35]. Changes in the levels of prostaglandins, growth factors, nitric oxide, cytokines and the amount of mucus may be involved in this type of lesion [36]. The results showed that, besides protecting the gastric mucosa against acute gastric lesions, treatment with EOHM at a dose of 400 mg/kg also speeded up healing of chronic ulcers in a manner comparable to pantoprazole. Moreover, according to histological analysis, rats treated with EOHM demonstrated the ability to regenerate the gastric mucosa (HE staining) and restore mucus production in glandular cells (PAS staining), as evidenced by the accumulation of pink in the layer of mucus cells not being observed in abundance in the internal area of the ulcer in animals from the control group. These results were confirmed by immunohistochemical analysis for PCNA, an important factor for healing of gastric mucosa, in which EOHM promoted an increase in cell proliferation in the region of regeneration.

This is the first report establishing the action mechanisms involved in the gastroprotective effects of the essential oil of *Hyptis martiusii* and its ulcer healing properties.

## Conclusions

The results suggest that the mechanism of action by which the essential oil of *Hyptis martiusii* protects the gastric mucosa can be partly attributed to its antisecretory properties, and partly to cytoprotective and antioxidant mechanisms, given their interaction with sulfhydryl compounds, resulting in an increase in gastric mucus, preventing depletion of non-protein sulfhydryl groups and reducing the levels of lipid peroxidation in the gastric mucosa. They also suggest that this essential oil is a promising candidate for the treatment of gastric disorders, in view its potential gastroprotective properties. However, we cannot affirm that a single sampling of the plant may be representative of the species as a whole.
